# The Nature of Age-Related Differences in Knee Function during Walking: Implication for the Development of Knee Osteoarthritis

**DOI:** 10.1371/journal.pone.0167352

**Published:** 2016-12-14

**Authors:** Katherine A. Boyer, Thomas P. Andriacchi

**Affiliations:** 1 Departments of Kinesiology and Mechanical and Industrial Engineering, University of Massachusetts-Amherst, Amherst MA, United States of America; 2 Departments of Mechanical Engineering and Orthopedic Surgery, Stanford University, Stanford, CA United States of America; 3 Palo Alto Veterans Affairs Health Care System, Palo Alto, CA, United States of America; University of Zaragoza, SPAIN

## Abstract

**Background:**

Changes in knee kinematics have been identified in the early stages of osteoarthritis (OA). However, there is a paucity of information on the nature of kinematic change that occur with aging prior to the development of OA, This study applied a robust statistical method (Principal Component Analysis) to test the hypothesis that coupling between primary (flexion) and secondary (anterior-posterior translation, internal-external rotation) joint motions in walking would differ for age groupings of healthy subjects.

**Methods:**

Seventy-four healthy participants divided into three groups with mean ages of 24 ± 2.3 years (younger), 48 ± 4.7years (middle-age) and 64 ± 2.4 years (older) were examined. Principal Component Analysis was used to characterize and statistically compare the patterns of knee joint movement and their relationships in walking.

**Results:**

There were significant differences between the younger group and both the middle-age and older groups in the knee frontal plane angle and the coupling between knee flexion (PC_1_, p≤0.04) and the relative magnitudes of secondary plane motions in early and late stance (PC_3_, p<0.01). Two additional principal components (PC_2_, p = 0.03 and PC_5_, p<0.01) described differences in early stance knee flexion and relationship with secondary plane motion through-out stance for the older compared with middle-age group.

**Conclusions:**

It appears there are changes in knee kinematics that occur with aging. The kinematic differences were identified for middle-aged as well as older adults suggesting midlife changes in neuromuscular physiology or behavior may have important consequences. These kinematic measures offer the potential to identify early markers for the risk of developing knee OA with aging.

## Introduction

Mobility is a major concern for older adults as it impairs activities of daily living and overall quality of life. In 2012, 23.1% of adults age 65+ reported difficulties with ambulation [1] and with the average population age continuously rising, this has the potential to become an important public health issue. Thus, it is crucial to understand factors that influence the maintenance of physical function from youth through old age. Age-related changes in muscle function and soft-tissue properties have been proposed as significant contributors to the decline in gait function, and thus mobility, in older adults [2]. In addition, osteoarthritic changes in joint tissue are a major factor in the decline of mobility with age. It is of importance that specific changes in knee kinematics be identified as they have the potential to influence the development of osteoarthritis (OA) and may contribute to further mobility loss [3]. The aim of this study was to examine impact of age on knee function during walking in individuals with healthy knees as it applies to the development of knee OA.

Independent of a slowing gait speed, older adult gait is often characterized by shorter stride lengths and a higher stride rate, or cadence, compared with younger adults[4;5]. Mechanical differences at the hip and ankle that underlie the spatio-temporal differences are relatively consistent in the literature. At matched speeds, older adults have increased hip range of motion, torque, power and work compared to younger adults [4–6]. Older adults typically show less range of motion at the ankle and have reduced plantar-flexion torque at push-off [4–6]. However, the effect of age on knee function during walking is less clear; preliminary literature suggests that older adults utilize a more flexed knee at heel-strike and a more vertically positioned shank when compared with younger adults [7;8]. Similarities between older adults, adults with early OA and individuals with a history of joint trauma (ACL) in the heel-strike knee flexion angle has led to the suggestion that heel-strike knee flexion may be a marker of OA risk [7].

Knee function is complex due to the shape and fit of the bony structures, and thus there is a strong coupling between the primary motion (knee flexion) and the secondary motions of the knee (internal-external rotation and anterior-posterior translation of the femur relative to the tibia) [9]. Muscle function can influence knee kinematics [10] and thus previously documented age-related changes in neuromuscular function could dynamically alter the kinematic coupling at the knee with aging [11]. Changes in the secondary plane knee joint kinematics following traumatic injury have been identified as markers of OA risk and connected with the initiation of degenerative changes in post-traumatic knee OA [3;12;13]. Most studies addressing the impact of age on joint function have focused on discrete time-point outcomes in a single plane of motion, ignoring the potential relationship between the planes of motion as well as the changes throughout time. Synergies or coordinative patterns describing the relationship between the joint degrees of freedom can be identified through dimensional reduction using principal component (PC) analysis methods. As PC analysis applied to a series of kinematic waveforms considers the overall behavior of the joint, it may provide a more comprehensive examination and allow for greater understanding of the differences in knee joint function with age.

While there are indications that movement mechanics do differ with age [2;4;8], most evidently in the sagittal plane, the relationship of sagittal plane kinematic changes with alterations in secondary motions of the knee remain unclear. A comprehensive examination of the effects of age on knee function may provide important information needed to better characterize the potential for kinematics to influence the development of idiopathic OA. Therefore, the purpose of this study was to test for a significant effect of age on knee function in the stance phase of walking. It was hypothesized there would be a progressive difference from younger, through middle, and up to older ages in the joint kinematics and kinematic coupling between the primary and secondary joint motions. The second aim of the study was to examine the nature of the changes in knee function with age, if present, through an in-depth examination of principal component vectors describing the primary directions of variance in the dataset.

## Methods

25 younger adults (24.0 ± 2.3 years; 23.0 ± 3.0 kg/m^2^; 11 female), 25 middle-aged adults (47.8 ± 4.7 years, 24.9 ± 3.6 kg/m^2^; 12 female) and 24 older adult participants (64.1± 2.4 years; 23.7 ± 3.8 kg/m^2^; 13 female) participated in this retrospective cohort study (Level III evidence). The study protocol was approved by the Stanford University Institutional Review Board and participants provided written informed consent prior to completion of any study activities. Participants were recruited from the community after responding to a print advertisement. Participants included in the analysis had a body mass index ≤ 30 kg/m^2^, no chronic lower body pain, and joints free of injury or cartilage pathology and severe mal-alignment. Joint health screening of both knees was performed using a 1.5T Magnetic Resonance (MR) System (GE Medical Systems, Milwaukee, WI). High contrast MR images were obtained via a fat suppressed three dimensional spoiled gradient recalled echo (SPGR) sequence in the sagittal plane using a standard transmits-receive extremity coil. The MR images were examined for signs of anterior cruciate ligament joint injury or repair and signal abnormalities indicative of cartilage pathology such as large defects or osteoarthritic changes (osteophyte presence).

Participants walked at self-selected preferred walking speeds while kinematic data for the left leg were collected using the Point Cluster Technique (PCT) marker set via an 8 camera high-speed infrared motion analysis system (ProReflex, Qualysis Inc, Sweden). Ground reaction forces were collected using a multi-component force-plate located in the center of the walkway (Bertec Corporation, Columbus, OH, USA). With the PCT markerset [14], clusters of nine and seven reflective markers are distributed on the thigh and shank, respectively, to estimate the movements of the underlying femur and tibia. Cluster coordinate systems are determined for the thigh and shank separately by calculating principal axes of the PCT marker clusters assuming a unit weight for each marker. During a static reference trial, markers placed on bony landmarks (medial and lateral malleoli, lateral and medial tibial plateau, lateral and medial femoral condyles, greater trochanters, anterior and posterior superior iliac spines, and the iliac crests) in addition to the marker clusters are used to establish the tibial, femoral and pelvic anatomic coordinate systems. The same research technician placed markers on all participants. The femoral anatomical coordinate system located at the midpoint of the transepicondylar line of the distal femur [14]. The longitudinal axis was defined as a vector from the hip joint center to the coordinate system origin, medial lateral axis was initially defined as a vector connecting the medial and lateral femoral epicondyle markers, and the third axis pointing anteriorly was perpendicular to the first two axes. The hip joint center was determined by the palpation method [15]. The anatomic tibial coordinate system was set at the midpoint of a line connecting the medial and lateral points of the tibial plateau [14]. The longitudinal axis was define as a vector connecting the origin to the midpoint of a line connecting the medial and lateral malleoli, the medial-lateral axis was defined by a vector connecting the medial and lateral points of the tibial plateau and the third axis was perpendicular to to these axis pointing anteriorly. The relative position between the marker clusters and the anatomical coordinate systems are calculated from the marker locations in the reference trial for the thigh and shank, separately. The knee flexion, ab-adduction and internal-external (IE) rotation angles and anterior-posterior (AP) translation of the tibia with respect to the femur were calculated as projection angles for the stance phase of gait [16]. AP translation of the tibia relative to the femur was determined by calculating the displacement of the origin of the tibial coordinate system relative to the projection of the femoral coordinate system onto the AP axis of the tibia. IE rotation of the tibia relative to the femur was calculated by projecting the medial-lateral femoral axis onto a plane created by the AP and medial-lateral axes fixed in the tibia. A zero angle or displacement was defined when axes or coordinate system origins were coincident.

A PC analysis was used to characterize and statistically compare the patterns of joint movement while also identifying interactions between the three components of joint rotation and the translation component [17]. A PC analysis will find the best low-dimensional representation of the variation in the 4-dimensional knee kinematics throughout the stance phase. The new variables (PCs) are a weighted combination of some or all of the knee kinematic data included in the analysis and represent the deviations in joint angle or displacement from the group mean. A PC that has more than one non-zero component represents variation in more than one waveform or percent of the gait cycle and identifies linear associations between planes of movement and phases of the gait cycle. PC analysis is scale sensitive and waveforms with a greater overall magnitude and range will be more heavily weighted. Therefore, standardization procedures were performed prior to completing the PC analysis to ensure equal weighting of each component of movement, while also retaining inter-participant and thus inter-group differences in the shape and timing of the waveforms[18]. This standardization ensures that measures such as the sagittal plane waveform with the largest range do not automatically infer greater biological relevance with regard to joint function or OA initiation.

Data analysis: For each step (foot-strike to toe-off), each of the kinematic waveforms was interpolated to 101 data points (0–100%) using a piecewise cubic hermite interpolating polynomial. The mean of each of the waveforms of interest was then calculated for each participant from 3 trials. The waveforms were standardized so that the mean of all trials was zero and there was unit variance across trials. The standardized waveforms for knee flexion, abduction, rotation and translation were then concatenated into a single data vector *V* with 404 vector components for each participant. The *V* vectors were combined into a single matrix (DATA [N x 404], where N = 74 participants) and the covariance matrix was computed. The eigen-vectors and values of this covariance matrix of the data were found. The eigenvectors, or PCs, are an orthogonal set of vectors that intersect at the mean of the trial vectors. The eigenvalues (EV) indicate the relative contributions of PC to the overall movement, expressed as the variance (%). Each participants’ trial vector was transformed into PC space via a simple coordinate system transformation to determine the PC scores (weightings). The PC scores represent the components of a vector (p-vector) locating each trial in the orthogonal coordinate system defined by the PC. All calculations were performed in Mathematica (Wolfram, Inc.).

Statistical Analysis: Two criteria were used to select the number of PCs to retain for hypothesis testing: 1) the *broken-stick* stopping rule [19], which was combined with 2) the proportion of total variance method [19]. With the *broken-stick rule*, a PC was considered non-trivial if its associated eigenvalue is larger than the value expected given a random distribution of the variance explain to all PCs. Of those non-trivial components, the number of PCs retained was such that 95% of the variance in the data set was explained.

To test for age-related differences in knee function, the relative positon in PC space of the trial vectors for middle-age and older participants were compared to those for the younger group. The length of a vector *(MC*_*dev*_*)* between p-vectors for each participant in the middle age and older groups and the mean p-vector for the younger group was calculated. A one-way ANOVA (p<0.05) was used to test the null hypothesis that *MC*_*dev*_ was not different from zero for the middle-age and older groups. An unpaired t-test was used to test for differences in *MC*_*de*v_ between the middle-age and older groups. A significant difference, from zero or between groups, in *MC*_*dev*_ would indicate that there is a change in the magnitude and pattern of knee kinematics with age.

While a main effect of age can be identified with the *MC*_*dev*_ metric, the direction or nature of these changes cannot be elucidated. The second aim of the study was to examine the nature of the changes in knee function with age, if present. A one-way ANOVA and a least significant difference post-hoc test with p<0.05 were used to test for age-group differences in the components of the p-vectors. To visualize and interpret the deviations from the mean represented by each PC, the data were plotted as the mean trial vector plus a PC vector weighted by the maximum and minimum score. In addition, for each group, comparison data were plotted as the mean trial vector plus a weighted linear combination of those PC that differed between the age-groups. This allows for visualization of only the variance from the mean that differentiates the groups.

## Results

**Fig 1:**
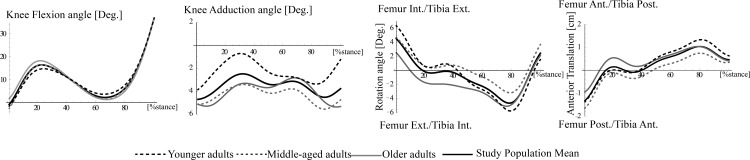
Mean stance phase joint kinematics for the 3 participant groups: younger (dashed black), middle-age (dashed grey) and older (solid grey). An overall study population mean of the stance phase kinematics is also illustrated solid thick black).

The first 7 non trivial PC summed together to explain 95% of the overall variance. The null hypothesis that the distance (*MC*_*dev*_) from the mean of the younger reference group was not different from zero was rejected in both cases (Middle-age *MC*_*dev*_ = 54.0 (SE 4.9), p<0.001; O *MC*_*de*v_ = 49.5 (SE 5.6), p<0.001). This indicates significant differences in the both the magnitude and pattern of the deviation from the overall mean in kinematics for the younger group, compared with the middle-age and older groups (Fig 1). There was not a difference in *MC*_*dev*_ between the Middle-age and older groups (p = 0.30). There were no significant differences in the self-selected normal walking speeds between age-groups (younger 1.33 (SE 0.04); middle aged 1.38 (SE 0.04); older 1.43 (SE 0.04)).

**Fig 2:**
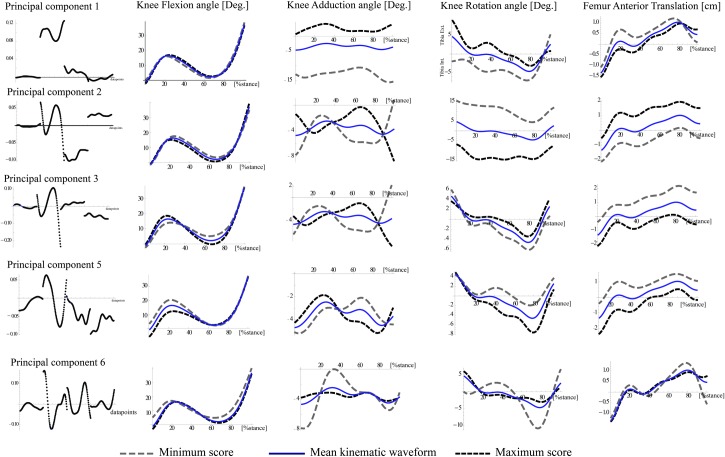
Waveforms were reconstructed using the maximum and minimum PC scores. The far left column illustrates the principal components that represent a specific pattern of deviation from the mean trial vector (i.e. waveforms) and the right four columns illustrate nature of those deviations from the mean waveform (solid line) based on the maximum and minimum PC scores. Our interpretation of the nature of these deviations is listed in Table 2.

The differences in knee function as compared to the younger group were not the same for the middle-age and older groups while PC scores were significantly different for five of the seven PCs examined (Table 1). Waveforms reconstructed using the maximum and minimum PC scores for each PC (Fig 2) were used to visualize the differences in knee function identified by each of the PCs. The first three PCs account for most of the variance in the kinematic waveform patterns between groups: PC_1_ loaded highly on components related to the ab/adduction angle and the early and mid-stance rotation and translation; PC_2_ loaded highly on the secondary joint motions and represented both local minima and maxima for the deviation in abduction angle in early and late stance, respectively; PC_3_ loaded on each of the 4 waveforms with varying magnitude for early and late stance (Table 2).

**Table 1:** The mean and SE of the PC scores that were significantly different between age-groups Younger (Y), Middle-age (MA) and Older (O).

PC Number	Eigenvalue (%)	Score Younger	Score Middle-age	Score Older	P-values
PC 1	65.9	16.5 (8.1)	-9.4 (8.1)	-7.4 (8.3)	Y-MA p = 0.03 Y-O p = 0.04
PC 2	16.3	-0.2 (4.1)	-6.2 (4.1)	6.6 (4.2)	MA-O p = 0.03
PC 3	4.4	-6.0 (2.0)	3.5 (2.0)	2.6 (2.1)	Y-MA p<0.01 Y-O p<0.01
PC 4	3.5	2.4 (1.9)	-0.21 (1.9)	-2.2 (1.9)	Y-O p = 0.1
PC 5	2.5	2.3 1.5)	1.7 (1.5)	-0.52 (1.2)	Y-O p<0.01 MA-O p<0.01
PC 6	1.5	-1.6 (1.2)	2.1 1.2)	-0.52 (1.2)	Y-MA p = 0.04
PC 7	1.2	-1.3 (0.9)	1.3(1.2)	-0.06(1.3)	

**Table 2:** The interpretation of those deviations from the mean knee function represented by each PC illustrated in Fig 2.

*Component*	*Interpretation*
*PC*_*1*_*- Overall Magnitude*:	A larger positive score (i.e. similar to younger group) indicated a change in frontal plane alignment towards a less abducted/more adducted position and a more externally rotated and anteriorly positioned tibia through to terminal stance. A large score also indicates a greater relative magnitude in adduction and external rotation in mid-stance.
*PC*_*2*_*-Flexion and secondary joint motion and coupling*:	A larger negative score (i.e. similar to middle-age group) indicates a more flexed knee in mid-stance and greater relative knee adduction in early versus later stance as well as a more externally rotated and anteriorly positioned tibia through-out stance.
*PC*_*3*_*- Relative early and late stance magnitude*:	A larger negative score (i.e. similar to younger group) indicated a smaller difference in the relative magnitude of the knee flexion and knee ab/adduction in early stance and late stance. In addition, a larger negative score corresponded with a more internally rotated and posteriorly positioned tibia relative to the femur.
*PC*_*5*_*-Early flexion and secondary motion coupling*:	A larger positive score (i.e. similar to middle-age group) indicates a more extended/less flexed knee at heel-strike and early mid-stance, greater knee adduction/less abduction in early mid-stance vs later mid-stance, and an internally rotated but more anteriorly positioned tibia in mid-stance through toe-off.
*PC*_*6*_*- Detailed features of the movement*:	This component describes the inflexions within curve including knee flexion and tibial external rotation at heel strike and local maxima in early stance for both the knee adduction and int/external tibia rotation.

**Fig 3:**
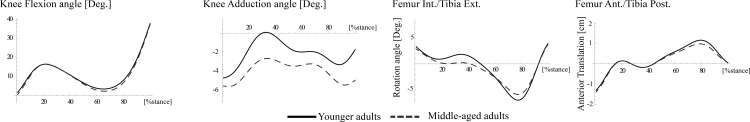
Data reconstructed for the young and middle aged groups using the average scores and PC1, PC3, and PC6. The middle-aged adults had a more abducted knee throughout stance, less tibia external rotation at heel strike and greater tibia internal rotation through middle and late stance, and greater tibia anterior translation in early stance but less anterior translation at terminal stance.

**Fig 4:**
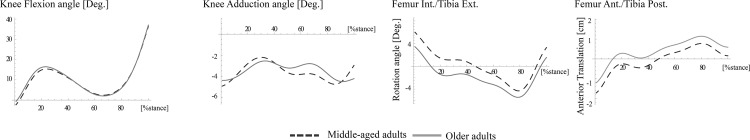
Data reconstructed using the average scores for PC2 and PC5. The older adults had less knee abduction in late stance and a smaller difference in the knee abduction magnitude between early and late stance local minimums. There was also a significant difference in the tibial internal-external rotation and AP translation throughout stance. The older adults tended to have more internally rotated and more anteriorly translated tibias relative to femurs throughout the stance phase.

To summarize the group differences, waveforms were also reconstructed using the mean trial vectors plus a weighted linear combination of those PCs that differed between the age-groups. The weighting coefficients were the mean scores for each PC (Figs 3 and 4). The overall differences for the middle-age group compared with the younger group (Fig 3) were a more abducted knee through-out stance, less tibial external rotation at heel-strike and greater tibial internal rotation through middle and late stance, and greater tibial anterior translation in early stance but less anterior translation at terminal stance. A comparison of the older group with the middle-age group indicated there were further differences with advanced age and a change in the coupling between the knee flexion motion and secondary plane motions and the relative magnitude of early and late stance knee ab/adduction motions (Fig 4). The older group also tended to have a more internally rotated and more anteriorly translated tibia relative to the femur through-out the stance phase.

## Discussion

The aim of this study was to test for a significant effect of age on knee function in the stance phase of walking. The finding that the distance in PC-space between the younger reference group and both the middle-aged and older groups was significantly different from zero provides evidence for a change in the magnitude and pattern of joint kinematics throughout stance with age. This result also suggests that changes in overall knee function during gait are evident in mid-life as well in later life. In the second aim, through an in-depth examination of principal component vectors describing the primary directions of variance in the dataset it was possible to identify unique kinematic features at the knee that change with age. These age-related differences in the kinematic waveform patterns were represented significant differences in the scores for five of the first seven PCs. Two of these PCs, accounting for almost 70% of the data variance, described differences for both the middle-aged and older groups in the frontal plane angle, the coupling between knee flexion, adduction and internal rotation and the relative magnitudes of the early and late stance kinematics. A significant difference in the scores for two other PCs, accounting for an additional 19% of data variance, indicated there were also differences between the middle-aged and older group in the change in early stance knee flexion and and the coupling between knee flexion and the secondary plane motion through-out the stance phase. These additional differences between the middle-age and older groups suggests there may be continual changes in knee kinematics and the kinematic coupling at the knee throughout the lifespan.

The primary differences from the young reference group for both the middle-age and older groups were found along PC_1_ and PC_3_. The middle-aged and older adults tended to have a more abducted, internally rotated and posteriorly positioned tibia relative to the femur during most of stance. This suggests a shift in the overall posture of the knee during walking in aging. This change in joint posture could result from a change in the static alignment at the knee. While, frontal plane standing leg alignment was not quantified in this study, a limitation of our work, data from the Copenhagen City Heart study suggests that there is an increased valgus alignment of the knee with age in non-OA knees [20]. However, the age-related kinematic differences identified in the present study were not limited to an offset in joint angle, the relative magnitude of the deviation from the mean between the early and later portions of stance, represented by PC_3_, also differed from the young group. For middle-aged and older adults, early stance was characterized by greater flexion, while the 2^nd^ half of the stance phase was characterized by less knee flexion. The local maxima and minima of the knee flexion-extension motion were coupled with the knee ab/adduction motion. The nature of this coupling was different across age groups. The younger adults exhibited less knee flexion and greater rotation towards adduction in early stance with a rotation towards abduction in later stance. The opposite was exhibited by middle-aged and older adults and greater knee flexion was accompanied by a reversal of the frontal plane knee motion, with abduction in early stance and adduction in later stance. The literature is inconsistent with respect to the abduction-adduction motion at the knee in walking. Some studies in agreement with the frontal plane motion for younger adults report a varus rotation in stance [14;21;22] while other studies report a valgus rotation [23;24]. Our results suggest that these discrepancies may be attributed to the age of the study population. Abduction or a valgus rotation can only occur with an active contribution of the local musculature as the external forces (i.e external adduction moment) would tend to force the knee into a varus posture through-out stance [10]. Changes in muscle function with age are well documented [11] and could be contributing factors along with passive tissue property changes to these differences in coupling between the primary and secondary plane knee function with age.

The second key finding in this study was the difference identified between the older and both the younger and middle-age groups along the 5^th^ PC. The 5^th^ PC represented both the variation in the early stance knee flexion and the coupling with the tibial abduction and anterior translation. The differences in the sagittal plane motion for the older compared to both younger and middle-aged groups, with a more flexed knee position at heel-strike and in early stance in older adults, were in agreement with the literature [6;25]. While PC_5_ explained only a small portion of the overall variance in knee kinematics, a secondary analysis indicated there were also significant group differences in the discrete time-point data for heel-strike knee flexion between the older and both the younger (mean difference 2.8 deg; p = 0.031) and middle-aged (mean difference 4.3 deg; p = 0.001) groups. The heel-strike knee flexion angle has been proposed as a mechanical marker of OA risk [7]. A more flexed posture, relative to controls, has been shown in trans-tibial-ACLR (at risk) [26], older asymptomatic (at risk), older early OA, and older end-stage OA groups [8]. Related to this change in knee flexion, but in contrast to what would be expected based on the proposed ‘screw-home’ mechanisms, a deviation in the tibial rotation at heel-strike and in early stance was not represented by PC_5_. Changes in the secondary kinematics that may come about due to the changes in coupling with knee flexion are important as they can alter the tissue strain patterns[12]. This alteration in knee coupling should be investigated further as a contributing factor to OA risk.

Previous research has focused on later-life adults, but only a few studies have examined the potential progression of changes in gait or joint function through the lifespan[4]. The results of this study suggest that changes in knee function occur much earlier than previously considered. The lifetime risk or probability of developing symptomatic knee OA increases substantially around 50 years of age [27]. The middle-aged group ranged in age from 40–54, and thus may be a group at high risk for development of OA. If gait mechanics are indeed related to increased OA risk as previously suggested [7], then these mechanical changes would be expected in emerge in middle-age, prior to or around the age of increased incidence in idiopathic OA. All participants in this study were screened via MRI for indication of cartilage abnormalities and thus at this time changes in knee function cannot be attributed to early structural changes indicative of early OA changes. The most likely explanation for these kinematic changes is a functional muscle adaptation such as increased muscle co-contraction as significant deficits in balance, proprioception and/or strength would not be expected in this middle-aged group. However, the potential for changes in balance, proprioception and strength during mid-life should be examined to understand how they may contribute to functional changes. A longitudinal cohort study is needed to quantify the underlying mechanisms for these changes and the impact of these age related changes in knee function on the OA risk.

Identifying differences in the secondary planes of motion between groups can be challenging due to the low signal to noise ratio. The overall patterns of motion in each plane (Fig 1) were consistent with the literature [9;28]. For all age groups there was an early stance flexion peak that was accompanied by a local maximum in the knee adduction and an inflection point in both the internal-external rotation and anterior-posterior translation of the tibia relative to the femur similar to previous reports [9;21;23;28]. To enhance our ability to detect significant age group differences in the magnitude and pattern of the secondary planes of motion at the knee, we used the PCT to calculated knee kinematics in combination with the PC analysis. The PCT is optimized for examining knee kinematics in situations that closely replicate natural movement [14]. The PCT method has undergone validation using in vivo testing, theoretical methods and through direct comparison with results from studies using more invasive methods such as bone pins or fluoroscopy [14;29]. Signal noise due to skin motion that may interfere with the ability to detect group kinematic differences is thought to be composed of random components as well as non-random components that can be related to the participant characteristics [30]. The PC retained for the analysis of age-related differences in gait had eigenvalues equal to or greater than 1% to minimize the potential for components representing the non-random noise to be interpreted as true differences in the underlying bones movement. In cross sectional studies if systematic differences in the groups exist that are not directly related to the physiological aging process the interpretation of the study outcomes can be compromised. While we carefully matched the groups for gender distribution, BMI and screened for joint pathology, in the present study we did not control for physical activity level. As decreases in activity level have previously reported in older adults [31] this could be a contributing factor to the changes in gait with age[4].

## Conclusion

While previous studies have identified differences between young and elderly gait at specific points of the gait cycle [2;6;8] the age-related changes in the patterns of movement have not been well characterized. Examining the full waveforms using PC analysis provided unique insight to the progressive changes not only in the magnitude of joint angles but also in the kinematic coupling (i.e screw home motion) at the knee with age. An investigation on the impact of changes in knee kinematics on the risk for OA initiation is needed.

## Supporting Information

S1 Table Individual PC scores for all participants and all PC (n = 74) are included in a supplementary data file. Each row contains data for a participants and each column is a PC score.
(XLSX)
